# AMPK, Mitochondrial Function, and Cardiovascular Disease

**DOI:** 10.3390/ijms21144987

**Published:** 2020-07-15

**Authors:** Shengnan Wu, Ming-Hui Zou

**Affiliations:** Center for Molecular and Translational Medicine, Georgia State University, Atlanta, GA 30303, USA; mzou@gsu.edu

**Keywords:** mitochondrial function, AMPK, cardiovascular disease

## Abstract

Adenosine monophosphate-activated protein kinase (AMPK) is in charge of numerous catabolic and anabolic signaling pathways to sustain appropriate intracellular adenosine triphosphate levels in response to energetic and/or cellular stress. In addition to its conventional roles as an intracellular energy switch or fuel gauge, emerging research has shown that AMPK is also a redox sensor and modulator, playing pivotal roles in maintaining cardiovascular processes and inhibiting disease progression. Pharmacological reagents, including statins, metformin, berberine, polyphenol, and resveratrol, all of which are widely used therapeutics for cardiovascular disorders, appear to deliver their protective/therapeutic effects partially via AMPK signaling modulation. The functions of AMPK during health and disease are far from clear. Accumulating studies have demonstrated crosstalk between AMPK and mitochondria, such as AMPK regulation of mitochondrial homeostasis and mitochondrial dysfunction causing abnormal AMPK activity. In this review, we begin with the description of AMPK structure and regulation, and then focus on the recent advances toward understanding how mitochondrial dysfunction controls AMPK and how AMPK, as a central mediator of the cellular response to energetic stress, maintains mitochondrial homeostasis. Finally, we systemically review how dysfunctional AMPK contributes to the initiation and progression of cardiovascular diseases via the impact on mitochondrial function.

## 1. Introduction

Cells constantly coordinate their metabolism to satisfy their energy needs and respond to the use of nutrients. Eukaryotes have developed a highly adaptive complex, the serine/threonine kinase adenosine monophosphate (AMP)-activated protein kinase (AMPK), to sense low cellular adenosine triphosphate (ATP) levels [1]. Under conditions of insufficient energy, AMPK activates upon its binding with AMP or adenosine diphosphate (ADP) [1]. Once activated, AMPK redirects metabolism toward increased catabolism or decreased anabolism through the phosphorylation of key proteins across multiple pathways, including protein synthesis (e.g., mammalian target of rapamycin complex (1) [2,3], lipid homeostasis (e.g., acetyl coenzyme A carboxylase, ACC) [4,5], glucose metabolism (e.g., phosphofructokinase (2) [6], and mitochondrial homeostasis (e.g., peroxisome proliferator-activated receptor gamma coactivator (PGC) 1 alpha, PGC1α) [7]. Besides, AMPK can adjust intracellular metabolism in a prolonged way by aiming at transcriptional regulators (e.g., forkhead box O_3_) [8]. Overall, activated AMPK balances energy levels by raising ATP synthesis and/or reducing ATP consumption. Thanks to its core roles in intracellular metabolism, dysregulation of AMPK is prevalent in obesity, diabetes, cancer, and cardio-metabolic diseases. AMPK is a prospective pharmacological target [9,10,11,12,13,14,15], notably for treating type 2 diabetes [16,17,18].

## 2. AMPK Structure and Regulation

### 2.1. AMPK Structure

AMPK, a heterotrimeric complex, is composed of a catalytic α-subunit and beta (β) and gamma (γ) regulatory subunits [19]. In humans, each subunit has multiple distinct isoforms encoded by different genes: the α-subunit has two isoforms, α1 and α2, encoded by gene *PRKAA1* and *PRKAA*2, respectively [20]; the β-subunit has two isoforms, β1 and β2, encoded by gene *PRKAB1* and *PRKAB2*, respectively [21]; and the γ-subunit has three isoforms, γ1, γ2, and γ3, encoded by gene *PRKAG1*, *PRKAG2*, and *PRKAG3*, respectively [22]. Each AMPK complex is comprised of an α-subunit, a β-subunit, and a γ-subunit, and all possible combinations produce 12 different AMPK complexes [23].

The α-subunit contains the kinase domain and a critical residue (Thr174 in α1-subunit and Thr172 in α2-subunit, hereafter referred to as Thr172) that is phosphorylated by upstream kinases [1]. The α-subunit is essential, and a double knockout (KO) of AMPKα1 and AMPKα2 in mice gives rise to embryonic lethality [24]. The β-subunit includes a carbohydrate-binding module that grants AMPK to bind to glycogen [25]. The γ-subunit allows AMPK to react to changes in the level of AMP, ADP, and ATP, as it includes four tandem cystathionine β synthase motifs that allow AMPK to bind adenine nucleotides [26].

### 2.2. Regulation of AMPK

#### 2.2.1. AMPK Is Activated by AMP/ADP and Inhibited by ATP

AMPK is activated by energy stress and/or cellular stress in response to increased ATP consumption (e.g., exercise, cell proliferation, and anabolism) or decreased ATP production (e.g., low glucose levels, oxidative stress, and hypoxia), which are sensed as low ratios of ATP-to-AMP/ADP. Binding of AMP, and to a lesser degree ADP, to the γ-subunit stimulates AMPK activity via three mechanisms [27,28,29]. First, AMP has been hypothesized to promote the phosphorylation of Thr172 by directly stimulating the activity of the upstream kinase, or by an allosteric mechanism that would render AMPK a suitable substrate for the upstream kinase [30,31,32]. Liver kinase B 1 (LKB1) [32] and calcium/calmodulin-dependent protein kinase kinase (CAMKK) 2 [33,34] can be employed as the two main mammalian upstream molecules that phosphorylate AMPK. Second, AMP protects Thr172 against dephosphorylation by phosphatases [28,35,36,37]. Third, AMP causes the allosteric activation of AMPK that has already been phosphorylated on Thr172. Activation loop phosphorylation increases AMPK activity by about 100-fold, while allosteric regulation changes AMPK activity up to tenfold in mammalian cells and about twofold in recombinant, bacterially produced AMPK [26,28,38,39]. Lastly, allosteric AMPK activation can also occur in non-phosphorylated AMPK, with even higher fold activation than phosphorylated AMPK [40].

#### 2.2.2. Regulation of AMPK by Reactive Oxygen Species (ROS) 

ROS are plenty of free radicals and chemically reactive molecules derived from oxygen molecule [41]. Most ROS are generated as byproducts of mitochondrial electron transportation from the reduction of molecular oxygen to produce superoxide [41]. The sequential reduction of superoxide gives rise to the generation of various ROS that includes hydroxyl radical, hydrogen peroxide, and hydroxyl ion [41]. The actions of ROS can be both beneficial and detrimental [41]. The beneficial aspects of ROS at physiological levels are usually related to their effects in cell survival signaling cascades [41]. Excessive production of ROS, usually termed oxidative stress, are caused by increased production and/or insufficient presence of endogenous antioxidants [41]. Oxidative stress is considered detrimental because of its damage to fats, deoxyribonucleic acid, and proteins in a cell, which can lead to numerous diseases such as diabetes, cancer, neurodegenerative disease, and cardiovascular disease (CVD) [41]. Recent lines of evidence have demonstrated AMPK responsiveness to imbalanced redox status, thus bringing new insights into the networks of redox-stimulated signals upstream of AMPK (Table 1) [42,43,44,45,46,47,48,49,50,51]. For instance, Trolox, a vitamin E analog and antioxidant, reduces cellular ROS levels [51]. The addition of Trolox to mouse embryonic fibroblasts (MEFs) resulted in basal AMPK activity reduction, as evidenced by the decreased phosphor-AMPKα at Thr172 [51].

##### Hydrogen Peroxide and AMPK Activation

AMPK is activated by hydrogen peroxide in various cell types. In H4IIEC3 cells, exogenous hydrogen peroxide transiently increased the level of phosphor-AMPK by LKB1 signaling, while exogenous hydrogen peroxide promoted AMPK phosphorylation in HeLa cells by CaMKK signaling [42]. Thromboxane A2 receptor (TPr) agonists increased the expression of nicotinamide adenine dinucleotide phosphate oxidase (NOX), thereby promoting the formation of superoxide in vascular smooth muscle cells (VSMCs) [52]. Exposure of cultured rat VSMCs to either TPr agonists or hydrogen peroxide led to AMPK activation in a time- and dose-dependent manner, as supported by the increased phosphor-AMPK at Thr172 and its downstream enzyme phosphor-ACC at Ser79 [61]. TPr agonists-activated AMPK was mediated by hydrogen peroxide, as demonstrated by the results showing overexpression of catalase (CAT) abolished Tpr agonists-induced AMPK activation [61]. In the meantime, LKB1 was activated by either TPr agonists or hydrogen peroxide [61]. Further, dominant negative mutation of LKB1 abolished both TPr agonists and hydrogen peroxide-stimulated AMPK activation, which suggested that hydrogen peroxide mediated TPr-triggered AMPK activation by LKB1 signaling [61]. These results collectively suggest that AMPK activation by hydrogen peroxide occurs through activation of either LKB1 or CaMKK, depending on the cell type.

ROS, in excess, can provoke energy stress and metabolic failure by direct oxidizing and inactivating enzymes of the tricarboxylic acid cycle, electron transport chain (ETC), and glycolysis [62,63]. It is well established that the ROS-induced energy stress strongly activates AMPK, which is likely the main mechanism of ROS-mediated AMPK activation. For instance, exposure of exogenous hydrogen peroxide to C2C12 cells led to a dose-dependent increase in AMPK activity by decreasing the ATP-to-ADP ratio [44]. Similarly, in NIH-3T3 cells, exogenous hydrogen peroxide transiently activated AMPK in a dose-dependent manner, which was associated with an increased AMP-to-ATP ratio [45]. Further, using HEK293 cells expressing AMPKγ2 R531G, an AMP-insensitive AMPK complex, AMPK cannot be activated by hydrogen peroxide [46]. These findings suggest that the target for hydrogen peroxide may not be AMPK itself, but components of the ETC, leading to a secondary effect on AMPK through changes to ADP, AMP, and ATP.

Alternatively, some studies showed that ROS directly activated AMPK independent of changes in ADP, AMP, and ATP. Exogenous or glucose oxidase-generated hydrogen peroxide induced direct S-glutathionylation on the AMPKα subunit at Cys299 and Cys304, which contributes to AMPK activation [48]. However, this view has been challenged with the fact showing that replacement of redox-sensitive cysteine residues with insensitive alanine residues does not affect hydrogen peroxide-induced AMPK activation [44,53].

##### Reactive Nitrogen Species (RNS) and AMPK Activation

Nitric oxide (NO) is produced by nitric oxide synthase (NOS) from l-arginine in the cell. RNS is produced beginning with the interaction between NO and superoxide to generate peroxynitrite. The sequential reaction of peroxynitrite with other molecules generates additional species of RNS, including dinitrogen trioxide and nitrogen dioxide, as well as other types of chemically reactive molecules. ROS broadly include NO and its derivative RNS, both of which have been implicated in AMPK activation.

Compared with wild-type (WT) mice, neuronal NOS KO (nNOS^−/−^) mice showed both reduction of stroke damage and AMPK activation, which suggests a possible link between NO and AMPK activity in the context of stroke [47]. In the studies using cultured bovine aortic endothelial cells (BAECs), peroxynitrite significantly activated AMPK, as supported by increased phosphor-AMPK at Thr172 as well as increased phosphor-ACC, a downstream target of AMPK, at Ser79 [54]. In endothelial cells (ECs), studies showed that NO endogenously activated AMPK via elevating Ca^2+^ levels by the activation of soluble guanylyl cyclase [55]. Further, NO-triggered Ca^2+^ increase causes AMPK activation through CaMKK2 signaling [55].

Exposure of ECs to chemically synthesized peroxynitrite acutely and significantly increased phosphorylation of AMPK and its downstream target ACC without affecting cellular AMP [56]. Hypoxia-reoxygenation (H/R) increased both AMPK and ACC phosphorylation in cultured BAECs, which was prevented by either inhibition of NOS or overexpression of SOD, suggesting a role of peroxynitrite formed during H/R [56]. These results indicate that H/R via peroxynitrite activates AMPK in ECs [56]. It is debatable whether LKB1 participates in AMPK activation under hypoxia condition. Some studies showed that LKB1^−/−^ MEFs under hypoxia for 30 min could not increase ACC phosphorylation [57], while LKB1^−/−^ MEFs under hypoxia for 2 h activated AMPK through CaMKK2 [58], indicating that LKB1-induced AMPK activation occurs in a context-dependent manner.

NO- and RNS-dependent activation of AMPK is probably related to the ability of NO and RNS to suppress mitochondrial ATP production by modulating the ETC [64]. In fact, NO at nanomolar concentration can form a nitrosyl–heme complex to selectively and reversibly inhibit cytochrome c oxidase [65]. RNS at higher concentration can induce cysteine S-nitrosylation or tyrosine nitration in almost all the complexes of mitochondrial ETC [66,67].

#### 2.2.3. AMPK Is a Key Modulator for Maintaining Redox Homeostasis

AMPK is not only a senor of redox signal, but also plays an anti-oxidative role once activated (Table 2). AMPK can diminish the superoxide production derived from either mitochondria or nicotinamide adenine dinucleotide phosphate oxidase (NOX). Further, AMPK also can suppress oxidative stress by upregulation of antioxidant gene expression. 

##### AMPK Suppresses ROS

Mitochondrial ROS is increased by many atherosclerosis stimuli, including hyperglycemia, triglycerides, and oxidized low-density lipoprotein (oxLDL) [74,75,76]. One study reported that pharmacological activation of AMPK by either salicylate or 5-aminoimidazole-4-carboxamide ribonucleotide (AICAR) regulated mitochondrial morphology and ameliorated endothelial dysfunction through suppression of mitochondrial ROS-associated endoplasmic reticulum (ER) stress and the subsequent activation of the thioredoxin-interacting protein-associated NACHT, LRR and PYD domains-containing protein 3 inflammasome [68]. Miglitol protected against endothelial cell damage from oxidative stress via inhibition of apoptosis and mitochondrial superoxide production by activation of the AMPK–eNOS axis [69].

Under basal conditions, in addition to mitochondrial ETC, NOX is another source of ROS production in ECs [77]. Inflammation-triggered endothelium activation generally gives rise to a large scale of ROS production that is mainly attributed to membrane-bound and/or cytosolic enzymes such as NOX [78]. Further, NOX-induced ROS production can enhance ROS production from other sources such as mitochondrial ETC and xanthine oxidase (XO) [77,79,80]. Analysis of AMPKα2 KO ECs and AMPK α2-silenced human umbilical vein endothelial cells (HUVECs) indicated that suppression of AMPK could increase NOX expression, NOX-mediated superoxide production, 26S proteasome activity, inhibitory subunit of nuclear factor kappa Bα degradation, and nuclear translocation of nuclear factor kappa B [70]. These studies indicated that AMPKα2 functions as a physiological suppressor of NOX and ROS production in ECs, in which way AMPK maintains the nonatherogenic and non-inflammatory phenotype of ECs [70].

##### AMPK Increases Antioxidant Potentials

The continuously produced ROS from mitochondrial ETC can be eliminated by antioxidant systems. It has been demonstrated that AMPK activation limited mitochondrial ROS generation in MEFs through PGC1α-dependent antioxidant response [51]. In this study, the potent AMPK activator A-769662 promoted an AMPK-dependent increase in several antioxidant genes, including *CAT*, superoxide dismutase *(SOD)1*, *SOD2*, and uncoupling protein 2 (*UCP2*). Moreover, a study using control or PGC1α-deficient MEFs with A-769662 showed that antioxidant gene expression was dependent on PGC1α expression. Similarly, in HUVECs, metformin normalized hyperglycemia-induced mitochondrial ROS production by inducing manganese SOD and promoting mitochondrial biogenesis through the activation of the AMPK–PGC1α pathway [71]. AMPK activation by either metformin or AICAR in HUVECs could suppress superoxide production by increasing the expression of the antioxidant gene *UCP2* [72]. Further, AMPK activation by metformin lowered the grade of acute lung injury and acute respiratory distress syndrome via the inhibition of oxidative stress by upregulating SOD1 and PGC1α, thus providing potential value for treating such conditions in clinic [73].

## 3. Status of Mitochondrial Function Controls AMPK

### 3.1. Mitochondrial ATP and AMPK Activity

Mitochondria are considered the primary source of ATP production in the cell. As AMPK is a low ATP sensor that restores intracellular ATP homeostasis, almost every mitochondrial insult, including respiratory chain complexes inhibitors (e.g., metformin, berberine, rotenone, and oligomycin) [46,81,82,83], proton ionophores (e.g., carbonyl cyanide-4-phenylhydrazone and dinitrophenol) [46], and mitochondrial deoxyribonucleic acid depletion or mutation [84], can induce AMPK activation. To this extent, AMPK serves as a mitochondrial guardian in an ancient and conserved role that occurs in multiple species including mammalian cells, yeasts, worms, and flies.

### 3.2. Mitochondrial ROS and AMPK Activation

Mitochondrial ROS can activate AMPK under both resting and stress conditions (Table 1). One of the components of mitochondrial complex III, Rieske iron-sulfur protein, has the ability to modulate mitochondrial ROS production [85]. Silencing Rieske iron-sulfur protein resulted in mitochondrial ROS production at resting condition, along with the phosphorylation of both AMPK and its downstream effectors unc-51 like autophagy activating kinase 1 (ULK1) and ACC in MEFs [51]. The AMP-to-ATP ratio between control and Rieske iron-sulfur protein-knockdown cells is comparable [51]. There is a line of evidence revealing that AMPK activation by mitochondria-generated ROS is essential for 2-deoxy-d-glucose-induced autophagy in cultured ECs [59]. 2-deoxy-d-glucose activated AMPK in ECs, which was inhibited by either antioxidants such as Tempol and N-Acetyl-l-cysteine or overexpression of antioxidant enzymes such as SOD1 and CAT [59]. Further, 2-deoxy-d-glucose-induced autophagy in BAECs was blocked by CAT overexpression or AMPK knockdown [59]. The participation of AMPK in ROS-triggered autophagy was considered to be critical for endothelial cell survival under stress conditions [59]. The involvement of mitochondrial-derived ROS in AMPK activation has been challenged by a study using a mitochondrial redox cycler MitoParaquat, which triggers a selective and continuous production of mitochondrial superoxide at complex I [86]. This study showed that mitochondria-generated ROS by MitoParaquat effectively change the thiol redox state of mitochondria in C2C12 cells without influencing AMPK activity, indicating that AMPK is unlikely be regulated by redox signaling in mitochondria [44].

Metformin has been reported to activate AMPK via mitochondrial-derived RNS production [60]. In cultured ECs, metformin activated AMPK, and likewise increased RNS production [60]. Further, either depletion of mitochondrial respiration or inhibition of NOS abolished the metformin-induced AMPK activation, implicating that metformin activates AMK likely via the mitochondria-generated RNS [60]. Consistently, administration of metformin led to activation of AMPK in the heart and aorta of WT mice, but not in those of eNOS-deficient mice [60]. Further, metformin greatly increased the interaction between AMPK and LKB1 in mice, which suggested that LKB1 was involved in metformin-induced AMPK activation [60].

Berberine improves insulin sensitivity in type 2 diabetes models via the activation of AMPK by inhibiting mitochondrial complex I, a similar mechanism to metformin [83]. Previous work from our laboratory has demonstrated that complex I-associated ROS production is related to many protective effects using metformin [60]. In fact, it has been identified that mitochondrial-derived ROS participate in the underlying mechanism of berberine-induced AMPK activation [43]. However, whether ROS generation attributes to the defect of complex I by berberine remains to be further explored. In cultured BAECs, berberine dose- and time-dependently activated AMPK, which was evidenced by the increased AMPK phosphorylation at Thr172 and its downstream substrate ACC phosphorylation at Ser79 [43]. Concomitantly, berberine induced production of peroxynitrite. Pretreatment of NOS inhibitor and overexpression of SOD could blunt berberine-induced AMPK activation [43]. Further, pretreatment of mitochondria-targeted antioxidant tempol or overexpression of uncoupling proteins (UCPs) suppressed berberine-induced AMPK activation [43]. Consistently, depletion of mitochondrial ETC abolished the effects of berberine on AMPK activation in ECs [43]. Berberine greatly activated LKB1 by increasing its phosphorylation at Ser307, and silencing of LKB1 suppressed berberine-induced phosphorylation of AMPK at Thr172 in BAECs [43]. These results indicate that mitochondria-generated superoxide and peroxynitrite are essential for berberine-triggered AMPK activation in ECs [43].

## 4. AMPK Regulates Mitochondrial Homeostasis

Mitochondria are vital organelles with multiple roles in modulating energy supply, ROS generation, and intracellular calcium dynamics. Mitochondria continuously change in number, size, shape, and intracellular location to maintain homeostasis. Mitochondrial dysfunction occurs in the development of many CVDs such as atherosclerosis, ischemia-reperfusion (I/R) injury, hypertension, diabetes, cardiac hypertrophy, and heart failure (HF), owing to the production of ROS beyond control. Therefore, early control of mitochondrial dysfunction is a promising step in CVD prevention and therapy. A growing body of evidence has demonstrated the specific regulation of various aspects of mitochondrial homeostasis by AMPK. These aspects include control of mitochondrial number through stimulation of mitochondrial biogenesis, regulation of the shape of the mitochondrial network, mitochondrial quality control through mitophagy regulation, and mitochondrial calcium influx. Here, we summarize the recent advances in the research on the regulation of mitochondrial homeostasis by AMPK.

### 4.1. AMPK Promotes Mitochondrial Biogenesis

Mitochondrial biogenesis occurs in response to increased energy expenditure (e.g., exercise) to produce more ATP. AMPK promotes mitochondrial biogenesis in various tissues, including myocytes [87], adipocytes [88], macrophages [89], and hepatocytes [90]. Accumulating studies have established that AMPK is a central regulator of mitochondrial biogenesis, with most studies focused on its effect in skeletal muscle [91]. For instance, β-guanadinopropionic acid-fed rats, in which skeletal muscle cells are in a state of chronic energy stress, showed chronic AMPK activation, increased nuclear respiratory factor 1 activity, and increased mitochondrial biogenesis in skeletal muscle [92]. Further, the AMPK-activating drug AICAR acted as an exercise mimetic to promote mitochondrial biogenesis in muscle [93]. Mice expressing a dominant-negative AMPK mutant failed to induce mitochondrial biogenesis in skeletal muscle under β-guanadinopropionic acid-induced energy stress [94]. The muscle-specific KO of AMPKα-subunit led to defects in mitochondrial biogenesis and function [87]. Mice lacking the AMPKβ1/β2 subunits showed a shortage of mitochondrial content in skeletal muscle [95]. Furthermore, overexpression of AMPKγ3 (R225Q) mutant, which increased basal AMPK activity in mice, induced mitochondrial biogenesis in glycolytic skeletal muscle [96], while muscle-specific LKB1 KO mice failed to increase mitochondrial biogenesis after exercise [97].

Most genes involved in mitochondrial metabolism seem to be under the control of the PGC1 family of coactivators, which is composed of PGC1α, PGC1β, and PGC1-related coactivator [98]. Overexpression of PGC1α in muscle is sufficient to convert type IIB (glycolytic) fibers into mitochondria-rich type II and type I fibers, which proves PGC1α as a master regulator of mitochondrial biogenesis [99]. Interestingly, it has been reported that the transcriptional upregulation of several genes is involved in oxidative metabolism, including glucose transporter 4, mitochondrial genes, and PGC1α itself, required PGC1α upon AMPK activation [7]. Overexpression of a constitutively active AMPKγ3 subunit increased PGC1α expression [96]. Two sites on PGC1α, Thr177 and Ser538, have been reported to be phosphorylated by AMPK both in vitro and in cells [7]. AMPK is also likely to regulate PGC1α by several indirect mechanisms involving the AMPK-dependent modulation of p38 mitogen-activated protein kinases [100], histone deacetylase 5-myocyte enhancer factor 2 signaling [101,102], and sirtuin (SIRT)1 [103]. These findings collectively suggest that AMPK communicates with PGC1α through a combination of mechanisms to regulate mitochondrial biogenesis. 

### 4.2. AMPK Regulates Mitochondrial Dynamics

Notably, some stimuli that activate AMPK also can induce mitochondrial fission, underlining a potential link between AMPK and mitochondrial fission. In fact, both rotenone (inhibitor of complex I) and antimycin A (inhibitor of complex III) can induce mitochondrial fission, which was mediated by AMPK activation [82]. Moreover, direct pharmacological activation of AMPK using A-769662 or AICAR, without mitochondrial damage, is able to trigger mitochondrial fission, establishing a role of AMPK in regulating mitochondrial dynamics [82]. Proteomic and bioinformatic screens have identified mitochondrial fission factor (MFF), one of the primary receptors of dynamin-like protein (DRP1) in the mitochondrial outer membrane, as a novel substrate of AMPK [82,104]. In human bone osteosarcoma epithelial cells (U2OS) and MEFs, AMPK directly phosphorylated MFF on two sites, Ser155 and Ser173 [82,104]. A further study showed that AMPK-driven MFF phosphorylation at Ser155 and Ser172 is one potential mechanism to account for mitochondrial fission owing to mitochondrial respiration decline [82]. Indeed, AMPK activation caused an increased mitochondrial translocation of DRP1, an effect that disappeared in AMPK-insensitive MFF mutants [82]. Thus, AMPK regulates mitochondrial dynamics during energy stress by the MFF–DRP1 axis [82].

Another study reported that AMPK modulated mitochondrial fission via a mechanism of autophagy-dependent degradation of DRP1 [105]. In mouse aortic endothelium, ablation of either AMPKα1 or AMPKα2 gave rise to impaired autophagy, DRP1 stabilization, and aberrant mitochondrial fission [105]. Furthermore, autophagy blockage by either chloroquine or autophagy-related 7 gene knockdown increased DRP1 protein levels and triggered DRP1-mediated mitochondrial fission [105]. Autophagy activation by mammalian target of rapamycin (mTOR) inhibitor rapamycin or autophagy-related 7 overexpression induced DRP1 degradation and diminished mitochondrial fission in AMPKα2-null mice, which suggests that impaired autophagy accounts for DRP1 accumulation and mitochondrial fission [105]. In addition, degradation of DRP1 in the autophagy–lysosome pathway was mediated by the binding of DRP1 with autophagic receptor protein sequestosome 1/p62, leading to the translocation of DRP1 into the autophagosome [105]. Further studies suggest that DRP1-dependent mitochondrial fission is responsible for endothelial dysfunction in AMPK-deficient mice [105].

### 4.3. AMPK Regulates Mitophagy

AMPK has been shown to regulate autophagy both in yeast [106] and in mammalian cells [107]. AMPK regulates autophagy through ULK1 via two different mechanisms. First, AMPK directly phosphorylates mTOR upstream regulator tuberous sclerosis complex 2 on Thr1227 and Ser1345 [3], and the subunit regulatory associated protein of mTOR complex 1 on Ser722 and Ser792 [2]. Both of these phosphorylation events contribute to reducing mTOR activity under energy stress conditions [2,3]. In turn, reduced mTOR activity removes the inhibitory phosphorylation on ULK1 to activate autophagy [108]. Second, AMPK directly phosphorylates ULK1 on four residues: Ser467, Ser555, Thr574, and Ser637 [109,110]. ULK1 phosphorylation by AMPK proved to be crucial for autophagy and cell survival during starvation and metabolic stress.

A series of later studies confirmed an essential role for ULK1 in mitophagy in various cells, such as brown adipocytes [111], myocytes [112], macrophages [113], and hepatocytes [114]. Strikingly, the AMPK–ULK1 axis is crucial not only for general autophagy, but also for mitophagy, as cells expressing ULK1 mutants resistant to AMPK phosphorylation accumulate defective mitochondria [110].

Inducers of mitophagy through the phosphatase and tensin homolog-induced putative kinase 1 (PINK1)– the E3 ubiquitin ligase (PARKIN) axis, such as the proton ionophore carbonylcyanide m-chlorophenylhydrazone [115], also strongly activate AMPK through the inhibition mitochondrial ATP synthesis [116]. Therefore, it is predictable that AMPK could be involved in the PINK1–PARKIN pathway. Indeed, a study using pressure overload-induced cardiac hypertrophy and HF models highlighted the role of AMPKα2 in the regulation of PINK1–PARKIN-dependent mitophagy [117]. It identified that, in the transverse aortic constriction (TAC) model, Ser495 in PINK1 underwent phosphorylation by AMPKα2 after dissipation of the mitochondrial inner membrane potential (∆Ψm) in cardiomyocytes [117]. This phosphorylation event was essential to efficiently recruit PARKIN to mitochondria and increase the rate of mitophagy during HF [117]. 

### 4.4. AMPK Regulates Mitochondrial Ca^2+^ Influx 

An appropriate amount of mitochondrial matrix Ca^2+^ is required for the activation of a number of enzymes of the mitochondrial ETC and tricarboxylic acid cycle, thereby maintaining mitochondrial respiration and ATP production [118,119,120]. It is well established that rapid Ca^2+^ influx into mitochondrial matrix occurs through the mitochondrial Ca^2+^ uniporter (MCU), a specific channel located at the inner mitochondrial membrane. However, the regulatory mechanism of this uniporter is still not clear [121,122,123]. The capacity of cells to alter bioenergetics in response to the demands of various biological processes is essential for normal physiology. MCU shows optimal performance in skeletal muscle and cardiac muscle, which experience large or acute demands in their energy utilization [124,125,126]. Cell division is also a highly energy-demanding cellular process, but the coordination of energy sensing and production is poorly understood. To this end, some study demonstrated a novel role for MCU-mediated mitochondrial Ca^2+^ transients in maintaining energy homeostasis during mitosis [127]. During mitosis, AMPK phosphorylated and activated MCU, allowing Ca^2+^ entry into mitochondrial matrix to enhance mitochondrial respiration [127]. These findings establish a critical role for the AMPK–MCU axis in regulating mitochondrial Ca^2+^ signaling that connects energy sensing to proper mitotic progression [127].

## 5. AMPK, Mitochondrial Function, and CVDs

CVDs include a number of diseases that involve the heart or blood vessels. CVDs represent the leading cause of mortality worldwide. AMPK is widely expressed in the cardiovascular system. The α1 isoform predominates in VSMCs, ECs, adipocytes, and monocytes/macrophages, whereas cardiomyocytes express much higher amounts of the α2 isoform. Numerous studies have provided proof for the concept that AMPK is protective across diverse cell types in the cardiovascular system. Moreover, aberrant AMPK signaling is involved in the pathogenesis of various CVDs, including hypertension, atherosclerosis, and stroke. Recent studies suggest that there is therapeutic value in targeting the AMPK signaling. Here, we briefly summarize the AMPK mechanism of action in the context of CVDs, and then focus on recent discoveries concerning the regulation of mitochondrial homeostasis by AMPK in CVDs (Table 3). A thorough understanding of the AMPK-mitochondrial axis in CVDs is an attractive target for therapeutically intervening CVDs.

### 5.1. Atherosclerosis

Atherosclerosis, a disease in which the inside of an artery narrows as a result to plaque build-up, is a leading cause of mortality and morbidity in the Western world. The formation of atherosclerotic plaques initiates with an insult to the endothelium (endothelial dysfunction), resulting in the deposition of lipids in the tunica intima. Subsequent oxidation and uptake of lipids by macrophages and VSMCs generate foam cells and, eventually, the characteristic plaque [143].

Endothelial dysfunction is considered a critical early event for atherosclerosis, ahead of angiographic or ultrasonic evidence of atherosclerotic plaques [144]. Damage to the endothelium unbalances vasoconstriction and vasodilation and initiates a number of events that promote or exacerbate atherosclerosis, including increased permeability of endothelium, aggregation of platelet, adhesion of leukocyte, and generation of cytokine [143].

Oxidative stress caused by risk factors represents a major contributor to endothelial dysfunction as a common predisposing condition to atherosclerosis. AMPK has been shown to confer potent antioxidant defense, which thereby contributes to vascular health [145].

AMPK activation improves the function of endothelium by defending oxidative stress in atherosclerosis, as supported by AMPK-regulated UCP2 expression in ECs. UCP2 presents in the inner mitochondrial membrane and belongs to the family of mitochondrial anion carrier proteins. It appears that UCP2 has a main function of controlling ROS generation in diabetes, obesity, and atherosclerosis, although it was originally considered as a modulator of nonshivering thermogenesis. The absence of UCP2 in mice has been reported to increase oxidative stress [146,147] and amplify the progress of atherosclerotic plaques [148]. Furthermore, emerging evidence shows that UCP2 is a modulator of mitochondria-generated ROS [149,150]. Berberine, a Chinese traditional medicine, suppressed oxidative stress and reduced atherogenesis by a mechanism that contains AMPK-dependent UCP2 expression mediated by nuclear respiratory factor 1 [128]. 

Additional evidence supporting AMPK protection against oxidative stress is that AMPK negatively regulates NOX by reducing its phosphorylation and preventing P47phox’s translocation to membrane [70,151]. AMPK suppresses protein kinase C activity and prevents protein kinase C modulation of p47phox phosphorylation [152]. Fucoxanthin, a natural marine carotenoid extracted from edible seaweeds, alleviated oxidative damage of ECs treated by oxLDL. The protective effects of fucoxanthin were attributed to activation of the AMPK-protein kinase C-NOX signaling pathway. Moreover, the suppression of oxidative stress, in turn, blunted the activation of the protein kinase B-cyclic AMP-responsive element binding protein–PGC1α pathway, which ameliorated mitochondrial dysfunction and apoptosis [129].

NO bioavailability is indicative of the NO production and utilization in endothelium. Its decrease occurs in response to lipid infiltration, oxidative stress, and inflammatory factors, which play a vital role in endothelial dysfunction [153]. Decreased NO bioavailability, manifested as impaired vasodilation, represents one of the early signs of atherosclerosis [154]. Obesity, diabetes mellitus, smoking, chronic kidney disease, and so on participate in the pathogenesis of atherosclerosis via a mechanism of changing NO bioavailability [153]. It is expected to achieve therapeutic significance in atherosclerosis via the strategies intending to enhance endothelial function by improving NO bioavailability [155]. Increasing evidence suggests that AMPK is essential in maintaining NO bioavailability. In response to several stimuli, including metformin [156], adiponectin [157], thrombin [158], histamine [159], berberine [160], ciglitazone [161], and vascular endothelial growth factor [162], AMPK is activated and in turn acts as a direct activator of eNOS by phosphorylating its Ser1177 residue [163]. Further evidence also shows that AMPK lies upstream of the phosphatidylinositol-3-kinase-protein kinase B pathway, which also leads to eNOS activation and NO production [162,164,165].

Some studies reveal the correlation of AMPK–eNOS signaling with mitochondrial function in ECs in the context of atherosclerosis. Salidroside, a phenylpropanoid glycoside isolated from the medicinal plant Rhodiola rosea, efficiently decreased atherosclerotic plaque formation in LDL receptor-deficient mice [130]. The main mechanism relied on the stimulation of eNOS activation and NO production through the AMPK-dependent activation of the phosphatidylinositol-3-kinase- protein kinase B pathway [130]. Salidroside-induced AMPK activation was presumably related to the moderate depolarization of ΔΨm and subsequent increase of the cytosolic AMP-to-ATP ratio [130].

Given the causative role of mitochondrial ROS in endothelial dysfunction in vascular diseases, it would be beneficial to either suppress mitochondrial ROS or promote mitochondrial biogenesis to compensate ROS-induced mitochondrial loss. Esculetin has been reported to protect HUVEC against cytotoxicity after exposure of linoleic acid hydroperoxide, which is likely via its radical scavenging ability [166]. Mitochondria-targeted esculetin, capable of scavenging mitochondrial ROS, greatly alleviated angiotensin II-induced monocyte infiltration, atherosclerotic plaque formation, and serum pro-inflammatory cytokine production in apolipoprotein E KO mice [131]. In cultured human aortic endothelial cells, mechanistic study showed that mitochondria-targeted esculetin significantly inhibited angiotensin II-induced cell death via enhancement of NO production via AMPK–eNOS signaling [131]. Notably, the anti-atherogenic effects of mitochondria-targeted esculetin are likely owing to the promotion of mitochondrial biogenesis through AMPK–SIRT3 signaling [131].

### 5.2. Ischemia

Ischemic vascular disease occurs when plaque builds up inside blood vessels and restricts normal blood flow. Angiogenesis is induced by ischemia and leads to new capillaries [167]. AMPK has been proven to be essential for ischemic angiogenesis; however, the mechanism by which AMPK promotes angiogenesis is poorly elucidated [165]. Study showed that inhibition of AMPK by either pharmacological inhibitor compound C or gene silencing significantly reduced tube formation in HUVECs [132]. Similarly, in comparison with the WT cells, tube formation in mouse arterial endothelial cells isolated from either AMPKα1^−/−^ or AMPKα2^−/−^ mice was significantly impaired, exhibiting oxidative stress and reduced expression of UCP2. Further, the impairment of tube formation in AMPKα1^−/−^ or AMPKα2^−/−^ cells could be normalized by adenoviral overexpression of UCP2 [132]. Alternatively, administration of NO donor, sodium nitroprusside, restored the tube formation in both AMPKα1^−/−^ and AMPKα2^−/−^ mice [132]. In comparison with WT mice, the ischemia-induced ser1177 phosphorylation of eNOS in thigh adductor muscles was blocked in both AMPKα1^−/−^ and AMPKα2^−/−^ mice [132]. Collectively, these results concluded that, in ECs, AMPK-dependent UCP2 expression together with eNOS phosphorylation promotes angiogenesis [132].

The heart is a highly aerobic organ [168]. Any condition of hypoxia may have a profound impact on cardiac function, and the majority of heart diseases are associated with cardiac hypoxia. According to the latest Global Burden of Disease Study, ischemic heart disease remained the leading global cause of death in 2017, accounting for 15.96% of total death worldwide [169]. Therefore, understanding molecular signaling transitions in cardiomyocytes under hypoxia may assist in the improvement of clinical treatments for heart disease.

In the heart, AMPK restores the ATP supply for cardiomyocytes and plays an essential role in both physiological states and stress conditions. Hypoxia activates AMPK through the ROS-mediated opening of calcium release-activated calcium channels [58,133]. A growing body of evidence has indicated that AMPK activation shows a protective effect on cardiomyocytes under myocardial ischemic injury [170]. Mechanically, AMPK increased glucose uptake and glucose transporter 4 translocation [171], decreased apoptosis, improved post-ischemic recovery, and limited myocardial infarction (MI) [172,173].

In cardiac muscle, chronic hypoxia induces oxygen-sensitive transcription to enhance carbohydrate metabolism, mitochondrial respiratory capacity, and the capability of mitochondrial energy supply. Thus, mitochondria were considered pivotal in cardiac regulation under hypoxia [174]. Under chronic hypoxia, PGC1α is upregulated, resulting in a subsequent increase of mitochondrial biogenesis [175]. However, with increasing mitochondria, the level of ROS is also increased as mitochondrial byproducts, which can result in oxidative damage of mitochondria [176]. Therefore, mitochondrial quality control is believed to be vital for maintaining the mitochondria in appropriate quality for cellular health. AMPK activation plays a crucial role in mitochondrial quality control under chronic hypoxia via modulating mitophagy in the heart [133]. Mitophagy increased significantly in cardiomyocytes exposed to hypoxic conditions, which was accompanied by AMPK activation [133]. Notably, AMPK agonist AICAR dramatically increased mitophagy [133]. Consistently, when AMPK activation was blocked, mitophagy decreased as well, which subsequently gave rise to cardiomyocyte apoptosis [133]. These results suggest that AMPK participates in mitophagy regulation, which thereby provides a valuable therapeutic target for ischemic heart diseases.

### 5.3. I/R

I/R injury happens when blood is resupplied to tissue (reperfusion) after a period of shortage (ischemia). The absence of oxygen and nutrients from circulation during the ischemic period establishes a condition in which the restoration of blood results in oxidative damage rather than the recovery of normal function. Despite the successful revascularization of occluded vessels in the heart, reperfusion failure occurs in approximately 30% of patients with acute MI [177,178]. I/R injury is primarily induced by endothelial cell apoptosis in microcirculation. The apoptosis induces endothelial swelling, microvascular spasms, and capillary obstruction, which slows or even stops microcirculation blood flow following reperfusion [179]. I/R injury causes myocardial damage and increases 30-day mortality rates [180]. Vasculature I/R injury alleviates the efficiency of reperfusion therapy and compromises the clinical benefits of patients with acute MI [181]. Therefore, strategies to alleviate microvascular damage from I/R may increase crucial adjuvant modalities for patients. Carvedilol, a third-generation β blocker, is in clinical use for heart failure patients. Carvedilol modulates cardiac AMPK signaling to reduce ischemic insults by I/R [182]. Treatment of cardiomyocytes with carvedilol augmented phosphorylation of AMPK and its downstream substrate ACC, and ameliorated hypoxia-induced impairment [182]. Importantly, carvedilol treatment improved calcium homeostasis with rescuing the peak Ca^2+^ signal, the maximum rate of Ca^2+^ change during contraction and relaxation under hypoxia conditions [182].

Recent studies suggest that the cardioprotective effects of metformin on I/R are mediated by its activation of AMPK. Administration of metformin before ischemia or at reperfusion decreased myocardial injury in mice [183]. During early reperfusion, treatment with metformin augmented I/R-induced AMPK activation and significantly increased eNOS phosphorylation at ser1177 [183]. The beneficial actions of metformin disappeared in cardiac-specific dominant-negative AMPKα2 transgenic mice or eNOS deficient mice [183]. Further, metformin failed to increase eNOS Ser1177 phosphorylation at reperfusion in AMPKα2 deficient mice [183]. These findings provide important information that myocardial AMPK–eNOS activation by metformin following I/R led to cardioprotection [183].

Recently, mitochondrial homeostasis has been attributed as a critical regulator of cell death during cardiac I/R injury, especially mitochondrial fission and mitophagy [184]. Excessive mitochondrial fission aggravates cardiac I/R injury via alternating the balance of pro- and anti-survival factors [184]. Mitophagy is an intracellular process of selective removal of severely defective mitochondria from the entire pool to maintain appropriate mitochondrial quality and homeostasis [185]. However, an excessive mitophagy leads to consumption of most mitochondria, which results in energy shortage and eventual cellular death.

The cardiac microvascular system is primarily comprised by monolayer ECs, where blood supply and nutrient exchange occur in the heart. There are a few strategies to alleviate pathologies of microvascular I/R injury after percutaneous coronary intervention. Melatonin treatment reveals a protective role in cardiac microvasculature under I/R injury by activating AMPK [134]. Melatonin significantly decreased myocadiac infarction, improved cardiac function, normalized blood flow, and lowered microcirculation perfusion impairment [134]. Histological analysis revealed that administration of melatonin restored the endothelial barrier, promoted eNOS expression, decreased inflammatory cell infiltration, and lowered endothelial damage in cardiac microcirculation endothelial cells [134]. Notably, all the beneficial effects of melatonin on microvasculature were abolished in AMPKα null mice [134]. In vitro mechanism studies showed that I/R triggered a DRP1-dependent mitochondrial fragmentation, which subsequently led to oligomerization of voltage-dependent anion channel 1, liberation of hexokinase 2, opening of mitochondrial permeability transition pore, upregulation of PINK1–PARKIN signaling, and eventual mitophagy-mediated cell death [134]. Melatonin promoted cell survival via AMPKα activation, accompanied by phosphor-DRP1-S616 reduction and phosphor-DRP1-S637 elevation, which normalized mitochondrial fission [134]. The prevention of mitochondrial fission further restored the interaction between voltage-dependent anion channel 1 and hexokinase 2, stopped mitochondrial permeability transition pore opening, and PINK1–PARKIN activation, ultimately blunting cell death [134].

Nitrite is a dietary constituent that protects the heart against I/R injury in many animal models during both ischemia and preconditioning stage [186,187,188,189,190,191,192]. During ischemia, the protective effects of nitrite are attributed to the reduction of nitrite to NO by deoxygenated haem proteins. The action of nitrite in preconditioning stage is dependent on AMPK. In cultured rat H9c2 cardiomyoblasts treated with H/R, preconditioning with nitrite markedly attenuated cell death following hypoxia [135]. Further mechanism studies showed that nitrite activated protein kinase A, which in turn suppressed DRP1-dependent mitochondrial fission, augmented ΔΨm, and promoted superoxide production [135]. Moreover, ROS scavenging blocks AMPK activation and blunts nitrite-induced cardioprotection after H/R [135]. Notably, in vivo animal study showed consistent results that nitrite protected the heart against I/R injury through the activation of protein kinase A [135]. These data first demonstrated the connection between nitrite and mitochondrial morphology/function in normoxia, which is important for cardioprotection. The activation of AMPK signaling is required for the cardioprotective effect of nitrite in the preconditioning stage of I/R [135].

### 5.4. Vascular Calcification (VC)

VCs are mineral deposits on the walls of arteries and veins. VCs are common, but considered a potentially severe condition by increasing the risk of stroke and blood clots. Thus, it is necessary to study the pathogenesis of VCs and develop effective therapeutic strategies to treat VCs in the body.

The mitochondrial open reading frame of the 12S rRNA-c (MOTS-c) is a bioactive mitochondrial-derived peptide shown to activate the AMPK pathway and modulate metabolic homeostasis [193]. Study showed that MOTS-c treatment significantly attenuated VCs induced by vitamin D3 and nicotine [136]. In parallel, phosphorylated AMPK increased and the expression of endothelin B (ETB) and angiotensin II receptor type 1 (AT1) receptors decreased after MOTS-c treatment [136]. ETB and AT1 are reportedly involved in AMPK pathway by binding to their receptors [194,195]. These findings indicate that MOTS-c may act as an inhibitor of VCs via suppressing the expression of AT1 and ETB receptors, probably by activating AMPK signaling [136].

### 5.5. Neointimal Hyperplasia Formation

Neointimal hyperplasia develops from excessive migration and proliferation of VSMCs from the medial layer toward the arterial lumen. Neointimal hyperplasia narrows the luminal diameter, leading to decreased blood flow and recurrence of anginal symptoms [196]. Thus, neointimal hyperplasia presents a significant clinical problem in patients with vascular disease.

The translocator protein (TSPO), an 18 kDa protein located at the outer mitochondrial membrane, is involved in the aberrant VSMC proliferation after angioplasty [137]. In a model of balloon-injured carotid arteries, TSPO expression was dramatically increased in carotid arteries [136]. Supplementation of PK11195, a ligand of TSPO, markedly reduced neointima formation in carotid arteries after balloon injury, through the suppression of the phenotype switching of VSMC [137]. Furthermore, it was found that PK11195 activated AMPK in A10 cells in a dose-dependent manner. An AMPK-specific inhibitor, compound C, abolished PK11195-induced inhibition of the proliferation and migration of platelet-derived growth factor-BB-treated A10 cells [137]. These results indicate that TSPO regulates neointimal formation after vascular injury via stimulating VSMC proliferation and migration, which is likely dependent on AMPK suppression [137]. TSPO could thus be a novel target for treating multiple CVDs, especially atherosclerosis and restenosis [137].

### 5.6. Cardiac Hypertrophy

Cardiac hypertrophy is the abnormal enlargement or thickening of the cardiac muscle, resulting from increases in cardiomyocyte size. The loss of AMPK activity is believed to be pro-hypertrophic because lines of molecular signaling pathways that control cell growth are attenuated by AMPK activation [197,198,199]. In parallel, cardiac LKB1 null mice are associated with hypertrophy [200]. Furthermore, AMPK deficiency also contributes to cardiac hypertrophy induced by aging, neurohumoral activation, pressure overload, and MI [201]. Activation of AMPK by AICAR or metformin protects the heart from cardiac hypertrophy induced by aging and other stresses [202]. However, some pharmacological AMPK activators, such as MK-8722 [203], cause cardiac hypertrophy, which is considered the main side effect of pharmacological pan-AMPK activation [17].

SIRT5, a member of the nicotinamide adenine dinucleotide-dependent sirtuin family of protein deacetylases, removes posttranslational modifications including malonylation, glutarylation, and succinylation on lysine residues [204,205]. SIRT5, primarily localizing at the mitochondrial matrix, plays a vital role in mitochondrial function via increased mitochondrial nicotinamide adenine dinucleotide and ATP synthase activity [138]. SIRT5 deficiency attenuated ATP production from mitochondrial ETC, increased the AMP-to-ATP ratio, and in turn activated AMPK in both cultured cells and hearts under the conditions of energy stress [138]. Consistently, SIRT5 ablation attenuated cardiac hypertrophy and cardiac dysfunction in the TAC model, which is accompanied by the reduction of ATP, increase of AMP-to-ATP ratio, and enhancement of AMPK activity [138]. Thus, this study unveils a crucial role of SIRT5 in controlling cellular energy metabolism by AMPK activation upon energy stress, which further confirms the cardioprotective role of AMPK in left ventricular hypertrophy under the stress of pressure overload [138].

Accumulating evidence suggests that mitophagy impairment can induce cardiomyocyte death and HF [206,207,208,209]. The most studied mechanism for mitophagy in mammalian cells is mediated by the cytoplasmic E3 ubiquitin ligase PARKIN [210] and the mitochondrial membrane kinase PINK1 [211]. Recently, studies in pressure overload-induced cardiac hypertrophy and HF models highlight the role of AMPKα2 in the regulation of PINK1–PARKIN-dependent mitophagy [117]. In failing heart samples from patients or TAC mice, the isoform of AMPKα shifted from α2 to α1, which is associated with reduced mitophagy and dysfunctional mitochondria [117]. The adenovirus overexpression of AMPKα2 in hearts blocked TAC-induced chronic HF via promoting mitophagy and mitochondrial function [117]. In contrast, AMPKα2^−/−^ mice showed an exacerbated progression of TAC-induced HF through defected cardiac mitophagy [117]. Further, ser495 in PINK1 underwent phosphorylation by AMPKα2 upon the dissipation of the ΔΨm in cardiomyocytes [117]. This phosphorylation proved critical for the efficient recruitment of PARKIN to mitochondria to initiate mitophagy during HF [117].

## 6. Cardio-Metabolic Diseases

### 6.1. Diabetes

Diabetes is a group of metabolic disorders featured by hyperglycemia over a prolonged time. The increased prevalence of diabetes was related to many factors, including diet, sedentary lifestyle, obesity, and aging [212,213]. CVD, accounting for over half of diabetic patients, is a major inducible factor of mortality in diabetic patients [214]. Endothelial dysfunction, the initial step of CVDs, is considered as a core modulator of vascular impairment in diabetes [215]. Endothelial dysfunction is attributed to many causes, including oxidative stress, chronic inflammation, and endothelium activation, in diabetes, which exhibits excessive free fatty acid (FFA), insulin resistance, and/or hyperglycemia [216,217].

In diabetes, mitochondrial dysfunction induces and aggravates macrovascular complications [218,219]. A line of evidence indicates that mitochondrial dynamics plays a pivotal role in endothelial dysfunction during macrovascular complications [220,221]. Both the conditions of hyperlipidemia and hyperglycemia impair mitochondrial dynamics by shifting the balance to excessive mitochondrial fission, leading to mitochondrial ROS over production and ETC impairment [221,222,223]. Additionally, mitochondria are a susceptible target of oxidative damage, which causes a malicious circle of events in ECs [224,225,226]. Thus, correcting the imbalance in mitochondrial dynamics during diabetes-induced endothelial dysfunction could improve patient outcomes. DRP1 is a cytoplasmic guanosine-5′-triphosphatase that triggers mitochondrial division by binding with mitochondrial receptors. Studies showed that AMPK deletion promoted mitochondrial fragmentation in ECs by inhibiting the autophagy-dependent degradation of DRP1 [105]. Numerous anti-diabetic agents may improve endothelial function by targeting AMPK to impact mitochondrial dynamics [227]. Clusterin, which exerts beneficial effects in ECs under diabetic conditions (both type 1 and type 2), inhibits mitochondrial fragmentation while activating AMPK [228]. Empagliflozin exerted its protective roles via inhibition of AMPK-dependent mitochondrial fission [229]. Empagliflozin recovered the AMP-to-ATP ratio that is necessary for triggering AMPK activation [229]. In the meantime, DRP1-S616 phosphorylation was decreased, while DRP1-S637 phosphorylation was increased, ultimately leading to mitochondrial fission [229]. Vildagliptin improved mitochondrial dysfunction in diabetic ECs, possibly by blunting DRP1-dependent mitochondrial fission by inducing AMPK activation [230]. Therefore, attenuation of mitochondrial fission by means of activating AMPK may represent a promising approach for macrovascular complications in diabetic patients.

Diabetic cardiomyopathy (DCM), a severe complication of diabetes mellitus, is a descriptive terminology showing the presence of myocardial dysfunction without apparent clinical valvular disease, coronary artery disease, and other conventional cardiovascular risk factors like dyslipidemia and hypertension [231]. Despite the significance of this complication, the underlying mechanisms of DCM are still poorly understood [232].

The mitochondria-associated ER membranes (MAMs) are referred to as the contact points by which the ER communicates with mitochondria [233]. MAMs play a vital role in lipid transport, Ca^2+^ signaling, cell survival, and energy metabolism [234], and have been implicated in diseases including Alzheimer’s disease [235], cancer [236], lysosomal storage disease [237], diabetes mellitus [238,239], obesity, and metabolic disorders [240]. Recently, the role of MAMs in the initiation and progression of DCM has drawn attention. Study showed that mitochondrial outer membrane protein FUN14 domain containing 1 (FUNDC1) is essential for maintaining the MAM structure and ensuring appropriate Ca^2+^ transfer from the sarcoplasmic reticulum (i.e., ER in myocytes) to mitochondria in healthy hearts [241]. Further, cardiac-specific KO of FUNDC1 gave rise to cardiac dysfunction via damaging MAM formation. High glucose-driven inactivation of AMPK increased FUNDC1, resulting in aberrant MAM formation, mitochondrial Ca^2+^ increase, mitochondrial dysfunction, and cardiac dysfunction [139]. In addition, AMPK activation reversed DCM by suppressing high glucose-induced MAM formation, mitochondrial Ca^2+^ increase, and mitochondrial dysfunction in cultured mouse neonatal cardiomyocytes 139]. These findings illustrating the results of AMPK activation, likely via downregulation of FUNDC1-related MAMs, represent a new therapeutic avenue for treating DCM [139].

### 6.2. Inflammation

Vascular inflammation, featured by the excessive production of pro-inflammatory cytokines (e.g., tumor necrosis factor α and interleukin 1β), can be caused by blood flow disturbance, toxins, hyperlipidemia, and hyperglycemia. Vascular inflammation stimulates the production of chemoattractants and molecules such as monocyte chemotactic proteins and vascular cell adhesion molecule 1 in endothelium [242,243,244]. Recently, deep mechanistic studies into the cardiovascular protection of AMPK using endothelial or myelomonocytic cell-specific AMPK KO mice have revealed the potent anti-inflammatory properties of AMPK in hypertension [145].

Metformin has been proven as an anti-inflammatory, especially for the inflammation in diabetes and following postsurgical endotoxin [245,246,247]. Additionally, AICAR, an AMPK activator, plays anti-inflammatory roles in vascular cells both in vitro and in vivo [248,249,250]. Atheroprotective flow patterns often give rise to anti-inflammatory conditions of the endothelium. In the clinic, the pleiotropic effects of statins on vessels are mediated by its anti-inflammatory effect and its ability to improve NO bioavailability. As discussed previously, a similar general outcome of metformin, AICAR, atheroprotective flow, and statins on ECs can be found through AMPK activation [245,250,251]. Indeed, studies have uncovered that AMPK exerted its anti-inflammatory roles on the vasculature partially via the inhibition of adhesion molecules and chemoattractants [248,249,252,253] by regulating signal transducer and activator of transcription 3, of nuclear factor kappa B, and p300 pathways [254,255,256].

AMPK was involved in inflammation-induced mitochondrial dysfunction and endothelial dysfunction. For instance, melatonin protected HUVECs against lipopolysaccharide-mediated apoptosis [140]. Lipopolysaccharide triggered cytosolic calcium overload, and in turn upregulated XO, whose activity is calcium-dependent [140]. Upregulation of XO was accompanied by excessive ROS production, resulting in the Ser616 phosphorylation as well as the mitochondrial migration of DRP1 [140]. DRP1-dependent mitochondrial fission triggered mitochondrial apoptotic pathway, as shown by the caspase 9 cleavage, ΔΨm decline, cytochrome c leakage, as well as increased expression of proapoptotic proteins [140]. Importantly, melatonin activated AMPK, which was associated with the upregulation of sarcoplasmic/endoplasmic reticulum calcium ATPase 2a [140]. Further, melatonin-activated AMPK-sarcoplasmic/endoplasmic reticulum calcium ATPase 2a axis prevented calcium overload, XO-dependent ROS overproduction, DRP1-mediated mitochondrial fission, and apoptosis in lipopolysaccharide-treated cells [140]. Collectively, this study demonstrated that melatonin induced endothelial cell apoptosis through activation of the AMPK-sarcoplasmic/endoplasmic reticulum calcium ATPase 2a pathway under conditions of inflammation [140].

### 6.3. Obesity

Obesity, a leading cause of preventable death worldwide, is prevalent in both children and adults and serves as one of the most significant public health problems [257]. Obesity is a medical disorder in which the excessive accumulation of body fat contributes to adverse health effects. Obesity-related type 2 diabetes patients often have elevated plasma FFAs, because of either increased amounts of FFAs released from large amounts of adipose tissue or impaired FFA clearance [258]. Elevated levels of plasma FFAs upset insulin signaling and often cause systemic metabolic imbalance. Hyperinsulinemia is a direct risk factor for atherosclerosis and other CVDs. Elevated plasma FFAs also cause inflammation, further contributing to CVDs.

The EC lining inside vascular walls serves as an interface between the surrounding tissue and circulating blood. Elevated levels of plasma FFAs, owing to their high density, could directly impair endothelial NO production, which is dependent on endothelial phosphatidylinositol-3-kinase-eNOS signaling. Thus, under diabetic conditions, ECs often have reduced eNOS activity and diminished NO production [259]. FFAs also contribute to a higher production of ROS and inflammation in ECs [260]. Collectively, inflammation, NO bioavailability, insulin resistance, and oxLDL are the main participating factors in obesity-related endothelial dysfunction [261].

Corosolic acid (CRA) is a natural triterpenoid with antioxidative activity. Study showed that CRA alleviated endothelial inflammation in obesity by activating AMPK [141]. CRA suppressed DRP1 activation by inducing DRP1 phosphorylation at Ser637, thus inhibiting mitochondrial fission in ECs in response to palmitate (PA) treatment [141]. CRA activated AMPK in a LKB1-dependent manner, and AMPK silencing abrogated its inhibitory effect on DRP1 activation and mitochondrial fission [141]. Concurrently, CRA suppressed PA-induced NOX2 activation, which was revealed by the reduced migration of p47phox to cell membrane and inhibition of gp91phox expression [141]. DRP1 knockdown attenuated PA-induced NOX2 activation, and knockdown of DRP1 or NOX2 suppressed PA-induced NACHT, LRR, and PYD domains-containing protein 3 inflammatory effect and, in the meantime, strengthened the inhibitory effects of CRA [141]. These data collectively demonstrated that CRA prevented mitochondrial fission by regulating DRP1 phosphorylation at Ser637 in an AMPK-dependent manner, contributing to the blockage of NOX2-related oxidative stress and suppressing NACHT, LRR, and PYD domains-containing protein 3 inflammasome in the endothelium [141].

Obesity is closely associated with cardiac disorders, which is assumed to be a consequence of the adaption to an oversupply of high-fat substrates [262,263]. Increased body weight switches the substrate of the heart from fatty acid to sugar, resulting in fat accumulation around the heart and eventually inducing cardiac impairment and increasing the risk of MI [264,265]. Risk factors such as oxidative stress, ER stress, inflammation, and mitochondrial dysfunction owing to fat accumulation have been reported to be highly connected with obesity-induced cardiac metabolic disorders and impairment.

AMPK activity was observed to be suppressed by high-fat diet (HFD) in diverse tissues, including white heart, adipose tissue, and liver [266]. HFD-induced AMPK deficiency contributed to the enhanced cardiomyocyte death in myocardial ischemia [267]. The loss of AMPK activity exacerbated cardiac hypertrophy and dysfunctional contractility of HFD-fed mice [268]. C1q/tumor necrosis factor-related protein 9 exhibited anti-myocardial lipotoxicity effects and attenuated cardiac hypertrophy, likely via activation of the LKB1–AMPK signaling [269]. A direct functional connection between AMPK signaling and β-adrenergic responsiveness exists in the heart, and AMPK might be a valuable target to recover the decreased β-adrenergic responsive in the heart of obesity [270]. Given that AMPK activation has beneficial metabolic consequences for obesity, AMPK has emerged as a promising therapeutic target for diabetic patients. Two classes of anti-diabetic drugs, metformin and thiazolidinediones, can function at least partially through the activation of AMPK in liver and muscle.

A recent study of subjects with type 2 diabetes offered clear evidence of mitochondrial dysfunction in permeabilized right atrial cardiac fibers [271], suggesting improvement to mitochondrial function as a potential therapeutic strategy for obesity and its associated pathologies. Punicalagin, the major active component in pomegranate extract, protects against HFD-induced cardiac disorders by activating AMPK pathway and promoting mitochondrial biogenesis [142]. In Sprague Dawley rats, administration of pomegranate extract, composed of 40% punicalagin, effectively blocked HFD-induced cardiac accumulation of fat (e.g., cholesterol and triglycerides) and myocardial damage [142]. Concomitantly, pomegranate extract activated AMPK, which may be responsible for the intervention of mitochondrial loss through promoting mitochondrial biogenesis and the attenuation of oxidative stress by means of upregulating phase II enzymes in HFD hearts [142]. Cell culture study demonstrated that punicalagin was the main component of pomegranate extract that specifically activated AMPK in cardiomyocytes via quickly reducing the intracellular ATP-to-ADP ratio [142]. These findings collectively elucidated that punicalagin, by activating AMPK, prevented HFD-related cardiac disorders via modulating mitochondria as well as phase II enzymes [142].

## 7. Perspectives

It is well-accepted that AMPK activation plays protective roles across diverse CVDs. AMPK has emerged as a guardian of mitochondria because almost all mitochondrial insults can activate AMPK. However, the interplay within the AMPK–mitochondrial axis during the initiation and progression of CVDs is poorly investigated. Limited evidence has focused on mitochondrial dynamics, mitochondrial biogenesis, and mitophagy, the three common functions of mitochondria that have been studied by cell biologists. Far beyond our current knowledge are the structural and functional changes to mitochondria that may be amplified under disease conditions. Therefore, it would be advantageous for investigators in the field of CVDs to focus on observing, defining, and clarifying novel characteristics and properties of mitochondria, thus contributing to both cell biology and advancing disease treatments.

## Figures and Tables

**Table 1:** The type of reactive oxygen species (ROS) that activates adenosine monophosphate-activated protein kinase (AMPK). ATP, adenosine triphosphate; ADP, adenosine diphosphate; BAEC, bovine aortic endothelial cell; MEF, mouse embryonic fibroblast; VSMC, vascular smooth muscle cell; HUVEC, human umbilical vein endothelial cell; NOS, nitric oxide synthase; LKB1, liver kinase B 1; CAMKK2, calcium/calmodulin-dependent protein kinase kinase; PDK1, pyruvate dehydrogenase kinase 1; CRAC, calcium release-activated calcium.

Stimuli	ROS	Cell Types	Animal Strains	Mechanisms	Refs
N/A	Hydrogen peroxide	H4IIEC3	N/A	PKCζ-LKB1	[42]
N/A	Hydrogen peroxide	HeLa	N/A	CaMKK	[42]
Berberine	Mitochondrial ROS, peroxynitrite	BAEC	N/A	LKB1	[43]
N/A	Hydrogen peroxide	C2C12	N/A	ATP-to-ADP ratio	[44]
N/A	Hydrogen peroxide	NIH-3T3	N/A	AMP-to-ATP ratio	[45]
N/A	Hydrogen peroxide	HEK293	N/A	ADP-to-ATP ratio	[46]
N/A	NO, peroxynitrite	N/A	nNOS^−/−^ mice	N/A	[47]
N/A	Hydrogen peroxide	HEK293	C3Ga.Cg-Cat B/J mice	S-glutathionylation of Cys299 and Cys304 on the AMPKα subunit	[48]
N/A	Mitochondrial ROS	MEF	N/A	N/A	[51]
Trolox	Physiological ROS	MEF	N/A	N/A	[51]
Thromboxane receptor	Hydrogen peroxide	VSMC	N/A	LKB1	[52]
Glucose oxidase	Hydrogen peroxide	HEK293, HeLa	N/A	AMP-to-ATP ratio	[53]
N/A	Peroxynitrite	BAEC	N/A	N/A	[54]
Sodium nitroprusside	NO	HUVEC	N/A	sGC-Ca^2+^-CAMKK2 axis	[55]
Hypoxia-reoxygenation	Peroxynitrite	BAEC	N/A	cSRC-PI_3_K-PDK1	[56]
Hypoxia	Mitochondrial ROS	AMPK α1^−/−^2^−/−^MEF	N/A	LKB1	[57]
Hypoxia	Cellular ROS	143B, LKB1^−/−^MEF	N/A	CRAC-CAMKK2	[58]
2-Deoxy-d-glucose	Mitochondrial ROS	BAEC	N/A	N/A	[59]
Metformin	RNS	BAEC	eNOS^−/−^ mice	cSRC-PI_3_K-LKB1 axis	[60]

**Table 2:** Activation of AMPK suppresses oxidative stress. AICAR, 5-aminoimidazole-4-carboxamide ribonucleotide; WT, wild-type; SD, Sprague Dawley; SOD, superoxide dismutase; CAT, catalase; UCP, uncoupling protein; NOX, nicotinamide adenine dinucleotide phosphate oxidase; PGC, peroxisome proliferator-activated receptor gamma coactivator.

Stimuli	ROS	Cell Types	Animal Strains	Mechanisms	Refs
A-769662	Mitochondrial ROS	MEF	N/A	AMPK-PGC1α-CAT/SOD1/SOD2/UCP2 axis	[51]
Salicylate, AICAR	Mitochondrial ROS	RAEC, EA.hy926	WT SD rats	AMPK axis	[68]
Miglitol	N/A	bEnd.3	N/A	AMPK-eNOS axis	[69]
N/A	Intracellular ROS	HUVEC	AMPKα2^−/−^ mice	AMPK-NOX axis	[70]
Metformin, AICAR	Mitochondrial ROS	HUVEC	N/A	AMPK-PGC1α-MnSOD axis	[71]
Metformin, AICAR	Superoxide	HUVEC	AMPKα2^−/−^ mice	AMPK-UCP2 axis	[72]
Metformin	Oxidative stress	N/A	WT BALB/c mice	AMPK-PGC1/SOD1 axis	[73]

**Table 3:** AMPK, mitochondrial, cardiovascular diseases (CVDs), and complications. SIRT, sirtuin; APOE, apolipoprotein E; LDL, low-density lipoprotein; HFD, high-fat diet; PA, palmitate; TAC, transverse aortic constriction; DRP1, dynamin-like protein; PINK1, phosphatase and tensin homolog-induced putative kinase 1; FUNDC1, FUN14 domain containing 1; ETB, endothelin B; MAM, mitochondria-associated endoplasmic reticulum membrane.

Reagents	Animal Strains	Disease Models	Culture Cells	Upstream of AMPK	Downstream of AMPK	Intracellular Effects	Cellular Effects	CVDs and Complications	Refs
Berberine	APOE^−/−^ mice, APOE^−/−^/AMPKα2^−/−^ mice	WD	HUVEC	Mitochondrial ROS	NRF1-UCP2 axis	Oxidative stress	Endothelial dysfunction	Atherosclerosis	[128]
Fucoxanthin	N/A	oxLDL	HUVEC	N/A	PKC-NOX-AKT-CREB-PGC1α axis	Oxidative stress; Mitochondrial dysfunction	Endothelial dysfunction	Atherosclerosis	[129]
Salidroside	LDLR^−/−^ mice	HFD	HUVEC	AMP-to-ATP ratio	PI3K/AKT-eNOS-NO axis	NO bioavailability	Endothelial dysfunction	Atherosclerosis	[130]
Mitochondria-targeted esculetin	APOE^−/−^ mice	AngII	HAEC	Mitochondrial ROS	eNOS-NO-SIRT3-TFAM/PGC1α axis	Mitochondrial biogenesis	Endothelial dysfunction	Atherosclerosis	[131]
N/A	AMPKα1^−/−^ mice, AMPKα2^−/−^ mice	N/A	HUVEC, MAEC, PMEC	Mitochondrial ROS	UCP2	Oxidative stress	Angiogenesis	Ischemia	[132]
					eNOS-NO axis	NO bioavailability			
N/A		N/A	H9c2	Mitochondrial ROS	N/A	Mitophagy	Cardioprotection	Ischemia	[133]
Melatonin	AMPKα^−/−^ mice	N/A	CMEC	N/A	DRP1-VDAC1/HK2/MPTP-PINK1/PARKIN axis	Mitophagy	Cell death	Ischemia /reperfusion	[134]
Nitrite	WT mice	N/A	H9c2	PKA-DRP1-mitochondrial ROS axis	N/A	Mitochondrial fission	Cell death	Ischemia /reperfusion	[135]
The mitochondrial open reading frame of the 12S rRNA-c	WT mice	Vitamin D3, Nicotine	N/A	N/A	ETB and AT1	N/A	N/A	Vascular calcification	[136]
The translocator protein	WT mice	Balloon injury model, PDGF-BB	A10	N/A	N/A	N/A	VSMC proliferation and migration	Neointima formation	[137]
N/A	SIRT5^−/−^ mice	TAC model	HEK293T	SIRT5-AMP/ATP axis	N/A	N/A	Cardiac dysfunction	Cardiac hypertrophy	[138]
N/A	AMPKα2^−/−^ mice	TAC model	HEK293T	N/A	PINK1/PARKIN axis	Mitophagy	Cardiac dysfunction	Cardiac hypertrophy	[117]
N/A	AMPKα2^−/−^ mice	STZ	H9c2, Cardiomyocyte	N/A	FUNDC1-MAMs axis	Mitochondrial dysfunction	Cardiomyopathy	Diabetes	[139]
Melatonin	N/A	LPS	HUVEC	N/A	SERCA2a-Calcium-XO-ROS-DRP1 axis	Mitochondrial fission	Cell apoptosis	Inflammation	[140]
Corosolic acid	WT mice	PA	RAEC, HUVEC	LKB1	DRP1-NOX2-ROS-NLRP3 axis	Mitochondrial fission; Oxidative stress; Inflammation	Cell death	Obesity	[141]
Punicalagin	WT mice	HFD	Cardiomyocyte	ADP-to-ATP ratio	PGC1α	Mitochondrial biogenesis	Cardiac dysfunction	obesity	[142]
					NRF2-Phase II enzyme	Oxidative stress			

## Abbreviations

ACC, acetyl coenzyme A carboxylase; ADP, adenosine diphosphate; AICAR, 5-aminoimidazole-4-carboxamide ribonucleotide; AMP, adenosine monophosphate; AMPK, adenosine monophosphate-activated protein kinase; APOE, apolipoprotein E; AT1, angiotensin II receptor type 1; ATP, adenosine triphosphate; BAEC, bovine aortic endothelial cell; CaMKK, calcium/calmodulin-dependent protein kinase kinase; CAT, catalase; CRA, corosolic acid; CVD, cardiovascular disease; CRAC, calcium release-activated calcium; DCM, diabetic cardiomyopathy; DRP1, dynamin-like protein; EC, endothelial cell; ER, endoplasmic reticulum; ETB, endothelin B; ETC, electron transport chain; FFA, free fatty acid; FUNDC1, FUN14 domain containing 1; HF, heart failure; HFD, high-fat diet; HUVEC, human umbilical vein endothelial cell; I/R, ischemia-reperfusion; KO, knockout; LDL, low-density lipoprotein; LKB1, liver kinase B1; MAM, mitochondria-associated endoplasmic reticulum membrane; MEF, mouse embryonic fibroblast; MFF, mitochondrial fission factor; MI, myocardial infarction; MOTS-c, the mitochondrial open reading frame of the 12S rRNA-c; mTOR, mammalian target of rapamycin; NADPH, nicotinamide adenine dinucleotide phosphate; nNOS, neuronal nitric oxide synthase; NO, nitric oxide; NOS, nitric oxide synthase; NOX, nicotinamide adenine dinucleotide phosphate oxidase; OX, xanthine oxidase; oxLDL, oxidized low-density lipoprotein; PA, palmitate; PGC, peroxisome proliferator-activated receptor gamma coactivator; PINK1, phosphatase and tensin homolog-induced putative kinase 1; PDK1, pyruvate dehydrogenase kinase 1; RNS, reactive nitrogen species; ROS, reactive oxygen species; SIRT, sirtuin; SOD, superoxide dismutase; TAC, transverse aortic constriction; TPr, thromboxane A2 receptor; TSPO, the translocator protein; UCP, uncoupling protein; ULK1, unc-51 like autophagy activating kinase 1; VC, vascular calcification; VSMC, vascular smooth muscle cell; WT, wild-type; ΔΨm, mitochondrial inner membrane potential.
